# Xylazine Infusion during Equine Colic Anesthesia with Isoflurane and Lidocaine: A Retrospective Study

**DOI:** 10.3390/ani13182902

**Published:** 2023-09-13

**Authors:** Patricia Ruíz-López, Charlotte Cuypers, Stijn Schauvliege

**Affiliations:** Department of Large Animal Surgery, Anesthesia and Orthopedics, Faculty of Veterinary Medicine, University of Ghent, 9820 Merelbeke, Belgium; charlottem.cuypers@ugent.be

**Keywords:** anesthesia, colic, equine, horse, lidocaine, recovery, xylazine

## Abstract

**Simple Summary:**

Equine colic is a critical and painful illness. Xylazine provides analgesia; sedation and muscle relaxation; and improves anesthetic recoveries in healthy horses. These might be useful for anesthesia in colic patients. This study aimed to retrospectively evaluate the intraoperative effects, and the influence on recovery from anesthesia, of adding a xylazine infusion (group XL) to isoflurane and lidocaine infusion (group L) for anesthesia maintenance. Cardiovascular parameters, blood gas analyses, anesthetic requirements, and time during recovery were retrospectively studied. Minimal and average heart rate, hematocrit, ketamine requirements and days to discharge were significantly lower in group XL than in group L. Time to sternal and first attempt to stand were significantly longer in group XL than in group L. Group XL showed almost twice the number of ‘best possible’ recoveries and no horses with the worst score. The reductions in heart rate and hematocrit were considered clinically irrelevant. The time to finally stand remained similar in both groups. Xylazine infusion might provide a stable anesthesia since less animals required ketamine. Animals in this group went home sooner and none of them had a dangerous recovery. Xylazine infusion might be a good option for anesthesia maintenance in colic patients.

**Abstract:**

This retrospective study investigated the effect of a xylazine infusion on heart rate; mean arterial pressure; blood gases; anesthetic and dobutamine requirements; recovery quality and duration; percentage of death/survival; and days to die/discharge in horses after colic surgery under partial intravenous anesthesia with isoflurane and lidocaine infusion. Anesthetic records of equine colic surgery were reviewed from similar periods in 2020–2021 and 2021–2022. In both groups, after sedation with xylazine 0.7 mg/kg intravenously (IV) and induction with ketamine 2.2 mg/kg and midazolam 0.06 mg/kg IV, anesthesia was maintained with isoflurane and lidocaine (bolus 1.5 mg/kg IV, infusion 2 mg/kg/h). Group L (2020–2021, *n* = 45) received xylazine 0.2 mg/kg IV before recovery, group XL (2021–2022, *n* = 44) received xylazine 0.5 mg/kg/h IV intraoperatively. In group XL, minimal (*p* = 0.04) and average (*p* = 0.04) heart rate, intraoperative hematocrit (*p* = 0.001), minimal (*p* = 0.002) and maximal (*p* = 0.04) dobutamine administration rate, animals requiring ketamine top-ups (*p* = 0.04), and the number of days to discharge (*p* = 0.02), were significantly lower compared to group L. During recovery in group XL, the time to sternal recumbency (*p* = 0.03) and time to first attempt (*p* = 0.04) were significantly longer. This retrospective study suggests that a xylazine infusion may have beneficial effects on horses undergoing colic surgery. Further prospective studies are necessary.

## 1. Introduction

Equine colic is a complex pathology that not only includes cardiovascular, blood gas and electrolyte disturbances, but also causes extreme pain [1].

Modern equine anesthesia aims to achieve unconsciousness, muscle relaxation and antinociception. In this way, partial intravenous anesthesia has been described using ketamine, lidocaine, alpha-2 agonists and opioids [2,3].

Currently, lidocaine infusions are commonly used during colic surgeries. The administration of lidocaine during exploratory laparotomy was associated with an increased survival rate in horses [4]. Lidocaine has antinociceptive [5], anti-arrhythmic and anti-endotoxic [6] properties and it also helps to improve gastrointestinal function postoperatively [7]. Driessen (2005) [8] described a reduction of up to 25% of isoflurane minimal alveolar concentration (MAC) with lidocaine 1.5 mg/kg and 1.8 mg/kg/h intravenously (IV), without significant effects during recovery when the infusion was stopped at the time that the surgeon started to close the abdomen in colic patients. Later, it was shown that a medetomidine infusion provided a better recovery quality than a lidocaine infusion in ASA I and II horses [9]. 

Alpha-2 agonists are widely used [10,11] because of their sedative, analgesic and muscle relaxant effect, reducing the MAC of inhalant anesthetics in horses. One of the biggest advantages of alpha-2 agonists is the improvement of recovery quality [12], which is one of the most dangerous phases of the anesthesia in equine patients [13].

Xylazine is the oldest alpha-2 agonist used to provide sedation in animals [14]. A bolus of 0.5 mg/kg IV reduced the isoflurane MAC by 25% in healthy horses [15] and a xylazine infusion of 1 mg/kg/h decreased the need for cardiovascular support in non-colic horses [16]. A bolus of 0.5 mg/kg IV followed by an infusion of 1 mg/kg/h over 2, 4 and 6 h in healthy, awake horses caused a significant decrease in heart rate (HR) and an increment in mean arterial pressure (MAP) during the first 30 min after the bolus administration, without subsequently decreasing MAP under baseline values [17]. A similar xylazine infusion administered over 60 min in healthy isoflurane-anesthetized horses resulted in a decrease in cardiac output and oxygen delivery, without affecting measured intestinal oxygenation [18]. Xylazine also produced good-quality recoveries, but the time in sternal recumbency and time to stand were increased [19]. 

To the authors’ knowledge, the use of alpha-2 agonist infusions during equine colic anesthesia has not been investigated. Our hypothesis was that the addition of a xylazine infusion to a lidocaine infusion during equine colic anesthesia might decrease isoflurane, ketamine and dobutamine requirements and improve recovery quality. The aim of this study was to compare the effects of a lidocaine infusion, followed by a xylazine bolus at the end of the anesthesia, to those of a lidocaine infusion combined with a xylazine infusion during isoflurane maintenance on (1) heart rate, mean arterial pressure, blood gases, anesthetic and dobutamine requirements; (2) recovery quality and duration; and (3) to compare the percentage of death/survival and days to die/discharge in horses after colic surgery.

## 2. Materials and Methods

### 2.1. Animals

Animals included in this retrospective study were selected by reviewing the anesthetic records of the Department of Large Animal Surgery, Anesthesia and Orthopedics at the Faculty of Veterinary Medicine, University of Ghent. After 7 months (mid-October 2021 to mid-May 2022) of using xylazine infusion in our colic anesthesia protocol, the same 7 months of the previous period (mid-October 2020 to mid-May 2021) without xylazine infusion were reviewed. Exclusion criteria were animals younger than 1 year old, pregnancy, intraoperative euthanasia because of surgical reasons, use of any other sedative for anesthesia sedation, use of an anesthetic infusion other than xylazine and lidocaine, a xylazine or lidocaine infusion not started from the beginning, receiving opioids perioperatively, or incomplete registered data.

### 2.2. Anesthesia

All animals presented for colic surgery were sedated with xylazine (Sedaxylan^®^, Dechra, Bladel, The Netherlands) 0.7 mg/kg IV. Anesthesia was induced with ketamine (Ketamidor^®^, Ecuphar NV, Oostkamp, Belgium) 2.2 mg/kg IV and midazolam (Dormazolam^®^, Dechra Veterinary Products, Lille, Belgium) 0.06 mg/kg IV. In both groups, anesthesia was maintained with isoflurane (IsoFlo^®^, Zoetis, Louvain-la-Neuve, Belgium) and lidocaine (Lidor^®^, Ecuphar NV, Oostkamp, Belgium) 1.5 mg/kg IV bolus followed by a 2 mg/kg/h IV infusion. Group L received a bolus of xylazine 0.2 mg/kg IV before recovery. Group XL received xylazine 0.5 mg/kg/h IV intraoperatively. If the anesthetist considered it appropriate, a xylazine 0.2 mg/kg IV bolus before recovery was also administered in group XL. All animals received flunixin meglumine (Emdofluxin 50^®^, Emdoka bv, Hoogstraten, Belgium) 1.1 mg/kg, sodium penicillin (Penicilline^®^, Kela Pharma, Sint-Niklaas, Belgium) 20,000 IU/kg and gentamicin (Genta-Equine^®^, Franklin Pharmaceuticals Limited, Meath, Irland) 6.6 mg/kg IV before sedation. All horses were positioned in dorsal recumbency, received Lactated Ringer’s solution (Ringer Lactate^®^; Baxter Healthcare S/A, Opfikon, Switzerland) 10–20 mL/kg/h intraoperatively and were mechanically ventilated with a tidal volume of 10–15 mL/kg and a peak inspiratory pressure limit of 30 cmH_2_O, to maintain expiratory CO_2_ between 35 and 55 mmHg. A dobutamine (Dobutrexmylan^®^, Cenexi, Fontenay-Sous-Bois, France) infusion, a noradrenaline (Levophed^®^, Hospira Benelux, Genk, Belgium) bolus or infusion and/or colloids (Geloplasma^®^, Fresenius Kabi, Schelle, Belgium) were given, depending on the anesthetist decision to maintain MAP ≥ 70 mmHg. No standardization was performed due to the retrospective nature of the study. Ketamine 0.4 mg/kg IV was given when the animal had nystagmus or movement intraoperatively.

### 2.3. Data Registered

Age (years); body weight (kg); gender (female, male, gelding); breed; anesthesia time (from start to end of isoflurane) (minutes); HR (before anesthesia; minimal, maximal and average HR intraoperatively) (beats per minute) (average HR was determined using all the HR values registered during anesthesia); invasive MAP (intraoperative minimal, maximal, average) (mmHg) (average MAP was determined using all the MAP values registered during anesthesia); SpO_2_ (intraoperative) (%); lactate (mmol/L) and glucose (mg/dl) (before anesthesia); PaO_2_ and PaCO_2_ (intraoperatively) (mmHg); hematocrit (%), pH, base excess (mmol/L), bicarbonate (mmol/L), natrium (mmol/L), chloride (mmol/L), potassium (mmol/L), calcium (mmol/L) (before anesthesia and intraoperatively); average end-tidal isoflurane (average was determined using all the registered end-tidal isoflurane values during anesthesia) (%); dobutamine (yes or no; duration of administration (minutes); minimal, maximal and average dose per minute of administration, and average dose per minute of anesthesia) (dose is given as µg/kg/min) (average dobutamine was determined by multiplying the rate by the time during which each rate was used and dividing the total amount by the total time that it was administered or the total time of the anesthesia, as needed); noradrenaline (bolus yes or no, infusion yes or no), colloids (yes or no), ketamine (yes or no; number of boluses per animal; total dose (mg/kg) per animal). 

Colic type was classified as (1) small intestine: strangulating; (2) small intestine: non-strangulating; (3) large intestine: tympanic, displacement, obstipation; (4) large intestine: torsion, intussusception colon; (5) small and large intestine; and (6) other: gastric volvulus, diaphragmatic hernia.

For recovery, it was registered if the animal, at the end of anesthesia, received a xylazine bolus or not in group XL (all animals in group L were sedated for recovery as described previously); if recovery was assisted with head and tail ropes; the number of attempts to stand; and different times (in minutes): time to extubation (from end of isoflurane to extubation), time in lateral (from end of isoflurane to sternal), time in sternal (since the animal was in sternal until it stood up), time to first attempt (from end of isoflurane to first attempt to stand), time to stand (from end of isoflurane to finally stand up). Recovery quality was registered as: (1) best recovery possible with 1 attempt, little to no ataxia; (2) 1 or 2 attempts, some ataxia; (3) >2 attempts, quiet recovery; (4) >2 attempts, moderate recovery; (5) >2 attempts, excitation; and (6) very bad recovery, high risk of injury. Recovery times and quality were retrieved from the anesthetic files, as they were registered during the real-time recovery. 

To determine the number of animals that died or were discharged, as well as the timing of death or discharge, only the first anesthesia was considered if the animal was anesthetized twice. 

### 2.4. Statistical Analysis

Normality was assessed visually analyzing values for mean–mode–median, skewness, kurtosis, quantile–quantile (Q-Q) plots and the Shapiro–Wilk test. An independent samples *t*-test was applied on parametric data; the results are expressed as mean ± standard deviation. A Mann–Whitney U test was applied on non-parametric data; results are expressed as median (range). Chi-square or Fisher’s exact test were used as needed to test the independency of gender, breed, colic type, recovery score, percentage of animals that received dobutamine, ketamine, noradrenaline bolus, noradrenaline infusion, and animals that died or were discharged. To analyze the number of animals that died or were discharged and the days on which these occurred, only the first anesthesia was included. Alpha was set at 5% (IBM^®^ SPSS^®^ Statistics for Windows, version 27.0, IBM Co., Armonk, NY, USA).

## 3. Results

No differences were found for age, weight, gender and anesthesia time between group L and XL. The frequency of assisted and non-assisted recovery was not statistically compared between groups, since only 3 animals in group XL had non-assisted recoveries (Table 1). Breed distribution is presented in Table 2 and colic type distribution is presented in Table 3. Only 12 animals in group XL received a bolus of xylazine before recovery and all the animals in group L received a bolus of xylazine before recovery. 

**Figure 1:**
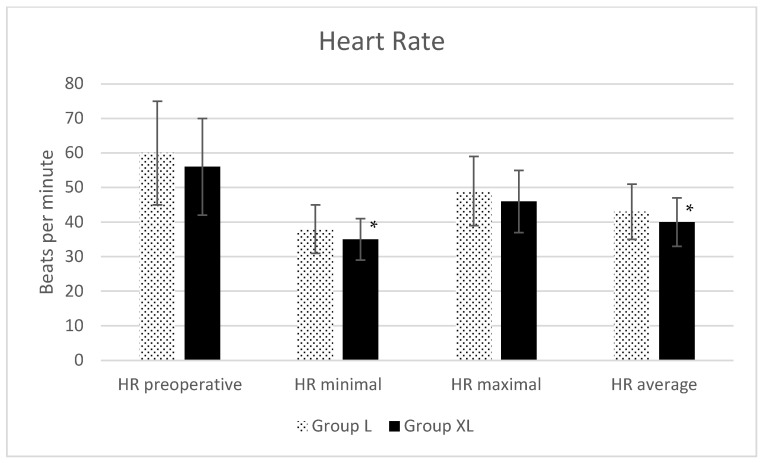
Preoperative and intraoperative minimal, maximal and average heart rates (HR) during isoflurane anesthesia for equine colic surgery. Group lidocaine (L) and group xylazine–lidocaine (XL). * Statistical difference (*p* < 0.05).

**Figure 2:**
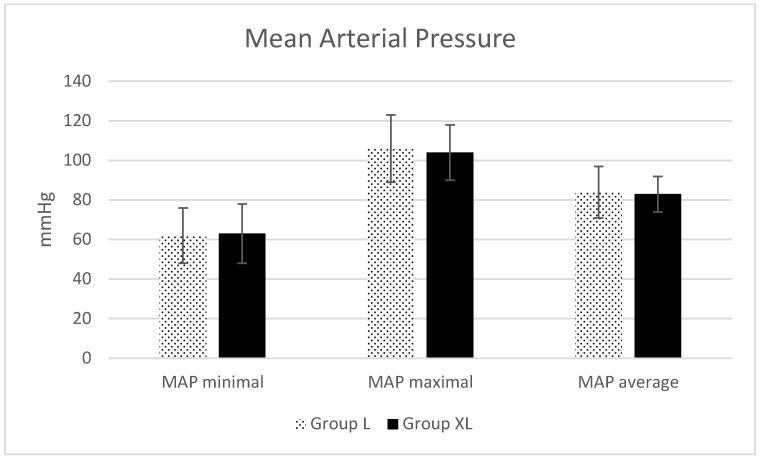
Invasive mean arterial pressure (MAP) minimal, maximal and average during isoflurane anesthesia for equine colic surgery. Group lidocaine (L) and group xylazine–lidocaine (XL).

**Figure 3:**
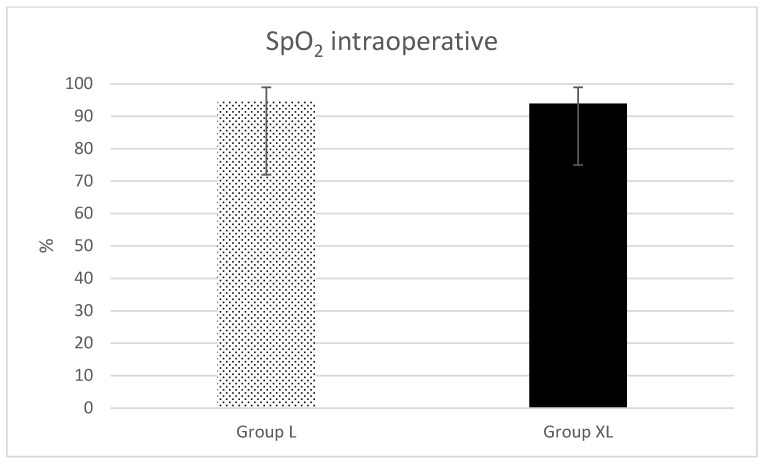
Peripheral oxygen saturation (SpO_2_) intraoperatively during isoflurane anesthesia for equine colic surgery. Group lidocaine (L) and group xylazine–lidocaine (XL).

Regarding HR (Figure 1), MAP (Figure 2) and intraoperative SpO_2_ (Figure 3), only statistical differences were found for minimal (*p* = 0.04) and average (*p* = 0.04) HR (Figure 1).

**Figure 4:**
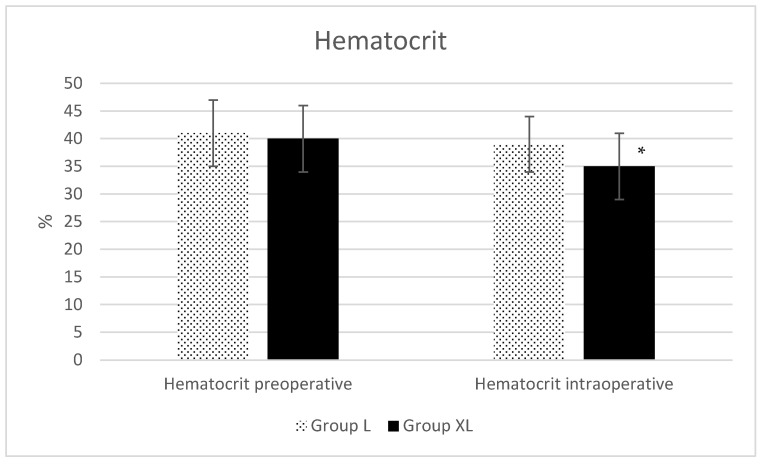
Preoperative and intraoperative hematocrit during isoflurane anesthesia for equine colic surgery. Group lidocaine (L) and group xylazine–lidocaine (XL). * Statistical difference (*p* < 0.05).

The blood gas analyses did not show any statistical differences. Data can be found as Supplementary Data (Table S1). Intraoperative hematocrit (Figure 4) was significantly lower (*p* = 0.001) in group XL.

No differences were found for the end-tidal percentage of isoflurane (Group L: 0.97 ± 0.13, Group XL: 0.93 ± 0.13, *p* = 0.09) or the percentage of animals that required dobutamine, noradrenaline boluses, noradrenaline infusion or colloids (Table 4).

**Figure 5:**
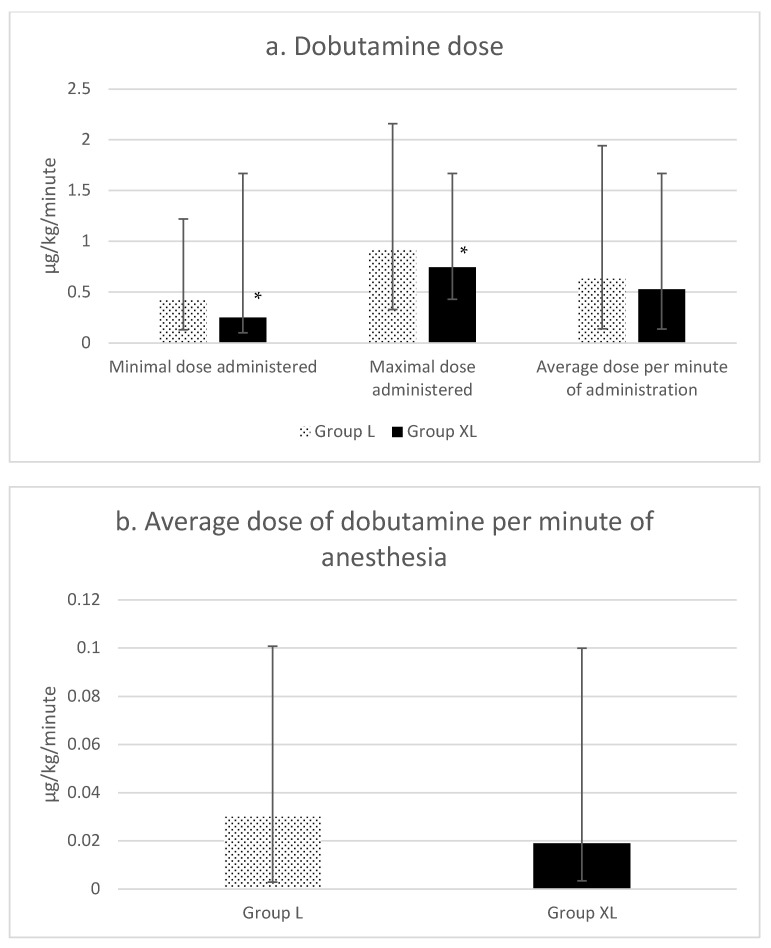
Figures represent: (**a**) minimal and maximal doses of dobutamine used, and average dose per minute of administration of dobutamine; and (**b**) average dose of dobutamine per minute of anesthesia during isoflurane anesthesia for equine colic surgery. Group lidocaine (L) and group xylazine–lidocaine (XL). * Statistical difference (*p* < 0.05).

There were no differences in the amount of time in which dobutamine was administered (Group L: 98 ± 46 min, Group XL: 85 ± 46 min, *p* = 0.23), in the average dose per minute of administration or in the average dose per minute of anesthesia (Figure 5). However, there were significant differences for the minimal (*p* = 0.002) and maximal (*p* = 0.041) doses of dobutamine used, which were lower in group XL (Figure 5a).

Significantly fewer horses received ketamine top-ups in group XL (Table 4). However, there were no differences in the number of boluses (group L: 1 (1–4), group XL: 1 (1–2), *p* = 0.33) or doses (mg/kg) (group L: 0.49 (0.16–2.25), group XL: 0.47 (0.11–1.4), *p* = 0.68) per animal that required ketamine.

There were 6 animals in group L and 1 animal in group XL with a second anesthesia. Eight animals from each group died. Percentages for death (group L: 20.5%, group XL: 18.6%, *p* = 1) and discharge (group L: 79.5%, group XL: 81.4%, *p* = 1) were similar between both groups. No differences were found for the day of death (group L: 5.5 (1–20) days, group XL: 3 (1–7) days, *p* = 0.23), but a statistical difference was found for the day of discharge (group L: 12 (5–40) days, group XL: 9 (5–52) days, *p* = 0.02).

Results regarding the different times analyzed for recovery are shown in Table 5. Significant differences were found for time in lateral and time to first attempt to stand. 

**Figure 6:**
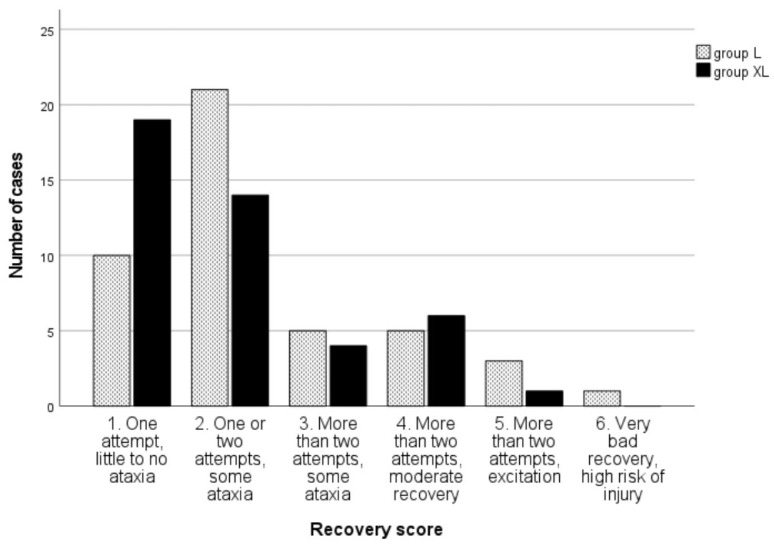
Number of cases by recovery score for groups lidocaine (L) and xylazine–lidocaine (XL) after isoflurane anesthesia for equine colic surgery, *p* = 0.26.

No differences were found regarding the quality of the recoveries (Figure 6).

## 4. Discussion

In this retrospective study on the influence of a xylazine infusion during the maintenance of anesthesia with isoflurane and lidocaine for colic surgery in horses, the intraoperative hematocrit; minimal and average HR; minimal and maximal dose of dobutamine; and days to go home were significantly lower in the group that received xylazine intraoperatively. The time in lateral recumbency and the time to first attempt to stand were significantly higher in the group receiving xylazine intraoperatively, but not the time to finally stand. In twice as many cases, the best possible recovery score was registered for group XL compared to group L, and none of the animals receiving xylazine intraoperatively received the worst possible score. 

Using alpha-2 agonists, such as xylazine, during equine colic anesthesia might be criticized due to the cardiovascular effects observed in some studies with healthy animals, where a decrease in cardiac output and oxygen delivery were shown [18]. However, the same authors pointed out that tissue oxygenation was maintained in healthy, well oxygenated horses. No differences (*p* = 0.49) in oxygenation between group L (PaO_2_: 132 (51–500) mmHg) and XL (PaO_2_: 152 (55–500) mmHg) were found in the current study. Cardiac output, oxygen delivery and tissue oxygenation could not be evaluated in this retrospective study. It was suggested that dexmedetomidine decreased oxygen consumption in isoflurane-anesthetized horses [20]. However, this has not yet been investigated with xylazine. 

The heart rate decreased and MAP increased in healthy horses for 30 min after giving a bolus and starting a xylazine infusion over 2, 4 and 6 h [17]. Reductions in minimal and average HR were indeed observed in the current study, but they might be considered clinically irrelevant since the minimal HR was 38 ± 7 beats per minute in group L and 35 ± 6 beats per minute in group XL, and the average HR was 43 ± 8 beats per minute in group L and 40 ± 7 beats per minute in group XL. No differences were found in MAP in this study between groups, independently of the treatment received. This could suggest that the clinically mild decrease in HR observed did not have a strong repercussion on the cardiovascular system of the animals in group XL. It is interesting to note that intraoperative tachycardia during abdominal surgery in horses has been related to an increased risk of mortality [21]. A decrease in HR might imply a decrease in cardiac output, but no cardiac output or other parameters with regard to cardiac output could be evaluated in this retrospective study.

Intraoperative hematocrit was significantly lower (*p* = 0.001) in group XL (35 ± 6%) compared to group L (39 ± 5%). This reduction could be attributed to intraoperative xylazine administration, since hematocrit reduction was found after the use of xylazine, detomidine and romifidine in horses [22,23,24]. The authors of the latter paper found an increased splenic thickness after a bolus administration of xylazine 0.5 mg/kg, romifidine 0.04 mg/kg and detomidine 0.01 mg/kg IV. In a similar way, xylazine 0.5 mg/kg IV caused a relative increase in spleen dimensions assessed sonographically using two long axes [25]. The most plausible explanation was a sequestration of the red blood cells in the spleen. In our case, this sequestration may have been maintained by the administration of a xylazine infusion that may have reduced the sympathetic tone as a result of the xylazine infusion itself or because of the improved analgesia. 

Requirements for cardiovascular support were decreased during the use of xylazine in healthy horses [16]. In a similar way, the administration of 1 mg/kg/h of xylazine in healthy animals resulted in a higher MAP in animals receiving xylazine compared with animals that did not [18] during the hour that the infusion was administered. However, all these animals received dobutamine 0.35 µg/kg/min during the maintenance of the anesthesia. In the current study, only minimal and maximal doses of dobutamine were lower in group XL than in group L. If the sample size had been calculated for the average dose of dobutamine per minute taking into account the results obtained in this study, 119 animals per group would have been necessary. Thus, increasing the number of animals might reveal significant differences in the average amounts of dobutamine required, as was already shown for the minimal and maximal doses; this should be considered in the planning of prospective studies. No differences were found for MAP, or in the percentage of animals that needed dobutamine, noradrenaline or colloids. No differences were found between the average dose of dobutamine per minute of administration or the dose of dobutamine per minute of anesthesia. Thus, a reduction in cardiovascular support was not proven. The lack of differences might be due to the retrospective nature of this study: the anesthetists were not blinded to the treatments, so it is difficult to determine whether (and to which extent) the use of a xylazine infusion influenced the concentration of isoflurane and the depth of anesthesia used in individual horses. Additionally, the similar amounts of cardiovascular support required in both groups might be explained by the pathophysiology of equine colic disease. Equine colic has severe repercussions on the cardiovascular system of the animals, and might present with dehydration, blood entrapment, preload decrease, endotoxemia, reperfusion shock, etc., resulting in high variations in cardiovascular function between cases. Additionally, the chosen dose in our study was 0.5 mg/kg/h, half the dose that showed an increase in MAP, but a decrease in cardiac output [17]. The xylazine infusion was given to improve the balance of the anesthetic colic protocol in our center. A relatively low dose was used, as previously described, in combination with other drugs in a balanced anesthetic protocol [26]. The dose of 0.5 mg/kg/h of xylazine is inside of the range of doses for xylazine infusion administration. Valverde (2013) [26] published a table with doses calculated (0.12 to 1 mg/kg/h) from available pharmacokinetic data to achieve therapeutic plasma concentrations of 340–800 ng/mL (partial intravenous anesthesia or standing sedation) with a clearance of 6 or 20 mL/kg/min.

Xylazine 0.5 mg/kg and 1 mg/kg IV had sparing effects on the isoflurane MAC in healthy horses with a baseline isoflurane MAC of 1.64 ± 0.05% [15]. However, the end-tidal isoflurane concentration was similar between group L (0.97 ± 0.13%) and XL (0.93 ± 0.13%) in the present study. It could be that further reductions were not possible since the end-tidal isoflurane concentration in group L was already lower than 1% and the clinical cases studied here presumably had high nociception levels because of the nature of the pathology—colic— and the surgical stimulation. Another plausible explanation might be the lack of the summative effects of lidocaine and xylazine infusions. The chosen dose of 0.5 mg/kg/h might also have been insufficient to show a summative effect with the lidocaine infusion at the end-tidal values found in this study. It would be useful to plan a prospective study where standardized increments and reductions in isoflurane are implemented and to include a group without lidocaine. Also, studies of the different doses of xylazine should be conducted. Nonetheless, the reduction in the percentage of animals that required an intraoperative ketamine bolus might indicate a more stable anesthetic plane or a better antinociception in group XL than in group L. 

Xylazine administration at the end of the recovery resulted in better recoveries of a longer duration in healthy horses compared to animals that did not receive xylazine [19]. No faster recoveries were observed after xylazine than with dexmedetomidine administration [27]. In this study, where all animals in group L and 12 of 44 animals in group XL received a xylazine bolus before recovery, no differences in the time to finally stand were observed between groups. However, the time in lateral recumbency and time to first attempt to stand were significantly higher in group XL. Recently, a xylazine infusion over 2, 4 and 6 h was not associated with an accumulation in the plasma or tissues of awake non-colic horses [17]. There are no data about the influence of anesthesia and colic surgery on the pharmacokinetic and pharmacodynamic properties of xylazine. We can assume that no significant accumulation occurred, as prolonged recoveries were not found in this study.

With regard to recovery quality, in group XL, almost twice as many animals as in group L received the best possible recovery score (one attempt with little to no ataxia), only one animal showed excitation with more than two attempts, compared with three animals in group L, and none of the animals in group XL showed the worst recovery possible, compared to one animal in group L. These findings might indicate that using a xylazine infusion has some advantages over using just a bolus before the recovery. 

It is also interesting to note that only 12 of the 44 horses in group XL received a bolus of xylazine for recovery. Xylazine vs. medetomidine infusion was studied in scheduled and non-scheduled equine general anesthesia by Kälin et al. (2021) [28]. They found that after including acepromazine at the premedication and giving a sedation of xylazine 0.3 mg/kg IV at recovery, 88% of the animals who received the xylazine infusion had a good recovery with 1 or ≥2 attempts to stand, but the animals remained calm. Our study only included emergency colic surgeries, none of them received acepromazine, and only 12 animals in group XL received an extra bolus of 0.2 mg/kg xylazine IV before recovery. Given the good recovery qualities in the current study, a bolus of xylazine before recovery may not be required in each individual colic patient when a xylazine infusion has been used.

No differences were found between death and discharge animals in the studied population, which may mean there were no deleterious effects of the xylazine infusion. We acknowledge the low number of animals included here, and we did not aim to perform a morbidity or mortality study. Nevertheless, while the day of death was similar for groups XL and L, the number of days to discharge was lower in the group XL, which might indicate some positive effects of the intraoperative xylazine infusion. This finding may be supported by a recent research study, where the authors found that preconditioning with xylazine 1 mg/kg/h IV during lidocaine 3 mg/kg/h might have a beneficial effect on equine jejunal ischemia and reperfusion injury [29].

The retrospective nature of this study is the main limitation, since no randomization or blindness could be applied and it was not standardized with respect to how isoflurane was to be reduced or incremented, or when ketamine, dobutamine, noradrenaline or colloids were to be given. A future study about intraoperative xylazine in equine colic anesthesia should include the monitoring of cardiac output, systemic vascular resistance, and oxygen delivery and consumption, which were not monitored in the current study due to its retrospective nature. The use of xylazine can lead to an overestimation of some of the results obtained with techniques such as the use of a lithium dilution cardiac output monitor [30], and this should be considered before selecting the technique. The unknown plasma concentration achieved in this study is another limitation. Plasma concentrations achieved with different xylazine infusion rates should also be considered in future studies on colic anesthesia. Another limitation is that different anesthetists with different degrees of experience were included. In addition, recovery scores may have been subjectively influenced [31]. The fact that 12 animals received a xylazine bolus in group XL for recovery is another limitation. The multiple underlying etiologies [32] that can cause colic in horses is another limitation, since a homogeneous population is difficult to achieve even if just one type of colic were to have been included. However, both groups presented similar types of colic, and there were no differences in the cardiorespiratory or blood gas analyses before the anesthesia. The study may have been underpowered as to what may have led to a type II error or false negative results; this should be considered when interpreting the results. Unfortunately, we cannot ensure that the sample population included in this study is a good representation of the entire population, since a sample size calculation was not performed before studying the parameters. 

It is common in clinical practice in different places to use alpha-2 agonists during colic anesthesia. Nevertheless, to the authors’ knowledge, no study looking at its use in this particular population has been published. Alpha-2 agonists have multiple properties, such as sedation, analgesia, and muscle relaxation that contribute to a balanced anesthesia. Colic patients might obtain the benefits of implementing alpha-2 agonists during their anesthesia.

## 5. Conclusions

A xylazine infusion during partial intravenous equine colic anesthesia with isoflurane and lidocaine was associated with fewer intraoperative ketamine boluses and earlier hospital discharge. It was associated with an increment in the time in lateral recumbency and a delay in the first attempt to stand, but not with a longer time to finally stand. Xylazine infusion during colic anesthesia with isoflurane and lidocaine was associated with almost twice as many excellent recoveries compared to the infusion of only lidocaine with a xylazine bolus for recovery. The intraoperative heart rate and hematocrit reduction were clinically irrelevant.

Balanced anesthesia and the administration of co-drugs has long been promoted, but today we realize that we do not know what the interactions are between these molecules, which is why we must be extremely cautious; observations concerning the administration of several molecules must be supported by strong evidence. Further prospective studies are warranted to confirm the results found in this retrospective study. 

## Figures and Tables

**Table 1:** Number of cases, age, weight, gender, assisted and non-assisted recovery and anesthesia times for group lidocaine (L) and xylazine–lidocaine (XL) during isoflurane anesthesia for equine colic surgery.

	Group L	Group XL	*p* Value
*n*	45	44	-
Age (years)	11 (1–27)	10.5 (1–27)	0.97
Weight (kg)	482 ± 130	482 ± 101	0.96
Male	6	3	0.44
Gelding	17	21	
Female	22	20	
Assisted recovery	45	41	0.15
Non-assisted recovery	0	3	
Anesthesia time (min)	125 (60–285)	115 (55–230)	0.31

**Table 2:** Breed distribution in group lidocaine (L) and xylazine–lidocaine (XL) during isoflurane anesthesia for equine colic surgery, *p* = 0.21.

Breed	Group L	Group XL
Anglo European Studbook	1	1
Arabian	1	3
Belgian Warmblood	16	18
Belgian Sport Horse	0	2
Connemara	3	0
Draft Horse	1	0
Friesian	0	1
German Warmblood	0	3
Irish Cob	1	0
Oldenburg	0	1
Pony	4	5
Quarter Horse	1	0
Royal Dutch Sport Horse	2	1
Selle Français	5	2
Spanish Warmblood	2	1
Standardbred	2	0
Thoroughbred	1	3
Zangerscheide	5	3

**Table 3:** Colic type distribution for group L (lidocaine) and group XL (xylazine–lidocaine) during isoflurane anesthesia for equine colic surgery, *p* = 0.69.

Colic Type	Group L	Group XL
Small intestine: strangulating	9	11
Small intestine: non-strangulating	3	6
Large intestine: tympanic/displacement/obstipation	16	17
Large intestine: torsion/intussusception colon	5	2
Small + Large intestine	10	7
Other: gastric volvulus/diaphragmatic hernia	2	2

**Table 4:** Percentage of animals that received ketamine, dobutamine, noradrenaline boluses, noradrenaline infusion and colloids during isoflurane anesthesia for equine colic surgery. Group lidocaine (L) and group xylazine–lidocaine (XL).

Medication	Group L	Group XL	*p* Value
Ketamine	57.77%	36.36%	0.04 ^1^
Dobutamine	95.55%	84.09%	0.09
Noradrenaline bolus	13.33%	11.36%	1
Noradrenaline infusion	31.11%	27.27%	0.69
Colloids	6.66%	4.54%	1

^1^ Significant difference, *p* < 0.05.

**Table 5:** Times (min) registered during recovery and number of attempts to stand for group lidocaine (L) and xylazine–lidocaine (XL) during isoflurane anesthesia for equine colic surgery.

Variable	Group L	Group XL	*p* Value
Extubation time	17 ± 9	19 ± 10	0.31
Time in lateral	30 (5–190)	37 (15–100)	0.03 ^1^
Time in sternal	1 (0–30)	1 (0–20)	0.63
Time to first attempt to stand	30 (10–190)	38 (15–92)	0.04 ^1^
Time to finally stand	35 (12–190)	44 (15–101)	0.06
Number of attempts to stand	2 (1–10)	1 (1–10)	0.64

^1^ Significant difference, *p* < 0.05.

## Data Availability

The data presented in this study are available on request from the corresponding author. The data are not publicly available due to confidential identification of the animals.

## Supplementary Materials

The following supporting information can be downloaded at: https://www.mdpi.com/article/10.3390/ani13182902/s1, Table S1: Preoperative and intraoperative blood gas values for group lidocaine (L) and group xylazine–lidocaine (XL) during isoflurane anesthesia for equine colic surgery.
