# Supplementary material for: Xylazine Infusion during Equine Colic Anesthesia with Isoflurane and Lidocaine: A Retrospective Study
Source: Animals (Basel). 2023 Sep 13;13(18):2902. doi: 10.3390/ani13182902 (PMC10525755; doi:10.3390/ani13182902)
Supplement: Supplementary file 1 [file animals-13-02902-s001.zip › animals-2555923-SI.pdf]

Blood gas values	Group L	Group XL	p value
Preoperative lactate (mmol/L)	1.89 (0.62–13.44)	1.76 (0.35–11.95)	0.95
Preoperative glucose (mg/dL)	132 (95-384)	129 (77–302)	0.73
Preoperative pH (venous)	7.4 ± 0.05	7.4 ± 0.05	0.81
Intraoperative pH (arterial)	7.35 ± 0.08	7.34 ± 0.06	0.71
Preoperative HCO_3^- (mmol/L)	28 ± 4.4	26 ± 4.6	0.19
Intraoperative HCO_3^- (mmol/L)	27 ± 4.1	27 ± 3.6	0.99
Intraoperative PaO_2 (mmHg)	132 (51–500)	152 (55–500)	0.49
Intraoperative $PaCO_2$ (mmHg)	51 ± 6.9	50 ± 6.4	0.52
Preoperative Natrium (mmol/L)	134 ± 3.1	133 ± 2.3	0.33
Intraoperative Natrium (mmol/L)	136 ± 3.7	135 ± 2.4	0.25
Preoperative Chloride (mmol/L)	96 ± 4.2	97 ± 3.3	0.25
Intraoperative Chloride (mmol/L)	97 ± 4.7	96 ± 4.3	0.46
Preoperative Calcium (mmol/L)	1.42 ± 0.09	1.42 ± 0.1	0.83
Intraoperative Calcium (mmol/L)	1.4 ± 0.12	1.38 ± 0.09	0.45
Preoperative Potassium (mmol/L)	3.51 (3.02–5.21)	3.61 (3.01–4.83)	0.77
Intraoperative Potassium (mmol/L)	3.36 (2.6–5.91)	3.23 (2.21–4.19)	0.76
Preoperative Base Excess (mmol/L)	3.3 (-7.6 – 9.6)	2.1 (-9.5 – 10.2)	0.23
Intraoperative Base Excess (mmol/L)	2.75 (-20.55 – 8.1)	1.15 (-9.24 – 7.05)	0.98

Table S1. Preoperative and intraoperative blood gas values for group lidocaine (L) and group xylazine-lidocaine (XL) during isoflurane anesthesia for equine colic surgery.
